# Influence of retinopathy on the achromatic and chromatic vision of patients with type 2 diabetes

**DOI:** 10.1186/1471-2415-14-104

**Published:** 2014-08-31

**Authors:** Luciana Cristina O Andrade, Givago S Souza, Eliza Maria CB Lacerda, Maira TST Nazima, Anderson R Rodrigues, Liudmila M Otero, Francineide PS Pena, Luiz Carlos L Silveira, Maria Izabel T Côrtes

**Affiliations:** 1 Programa de Pós-Graduação em Ciências da Saúde, Universidade Federal do Amapá, 68903-419 Macapá, Amapá, Brazil; 2 Instituto de Ciências Biológicas, Universidade Federal do Pará, Belém, Pará, Brazil; 3 Núcleo de Medicina Tropical, Universidade Federal do Pará, Belém, Pará, Brazil

**Keywords:** Type 2 diabetes, Visual psychophysics, Diabetic retinopathy, Contrast sensitivity, Colour vision, Farnsworth–Munsell test

## Abstract

**Background:**

Luminance contrast sensitivity and colour vision are considered to have great predictive value in the evaluation of type 2 diabetic retinopathy. However, these two visual characteristics have seldom been investigated in the same group of patients. In the present study we measured contrast sensitivity and colour vision in a group of patients with type 2 diabetes and correlated the results with estimates of common metabolic markers for the disease. A subgroup of the patients had no clinical signs of retinopathy.

**Methods:**

The vision of 27 patients (n = 50 eyes) with type 2 diabetes, with retinopathy (n = 20 eyes), or without retinopathy (n = 30 eyes) were evaluated using two psychophysical tests, the Farnsworth–Munsell 100 hue test (FM 100), and measurements of the luminance contrast sensitivity at 11 spatial frequencies. The results were compared with measurements obtained from an age-matched control group (n = 32), and were correlated with the level of glycated haemoglobin, glycaemic level, and time of disease onset. Signs of retinopathy were identified during the ophthalmological examinations.

**Results:**

Contrast sensitivity and colour vision impairments were present at different levels in diabetes patients. Eyes with retinopathy showed more severe vision loss than eyes without retinopathy. The FM 100 test was more sensitive for separation of patients from controls. Colour vision loss had no colour axes preference. The contrast sensitivity test appeared to have some advantage in differentiating patients with retinopathy from patients without retinopathy.

**Conclusions:**

Both methods can be useful to follow the visual function of diabetic patients and should be used together to discriminate patients from controls, as well as to identify early signs of retinal damage.

## Background

The World Health Organization predicts that by the year 2030, 350 million people will be suffering from type 2 diabetes, and their care will account for up to 15% of the funds allocated to national healthcare [1,2]. Type 2 diabetes is a prominent cause of acquired blindness or severe visual impairment [3,4], and diabetic microvascular complications affecting the retina lead to a form of retinopathy that could be very detrimental to a patient’s life [5-7]. These complications generally increase the risk of visual loss by impairing several aspects of visual functions [8].

Diabetic retinopathy is characterized by progressive changes in the structure of the retinal microvasculature. It can lead to the development of micro aneurysms, haemorrhages, ischaemia, and poor retinal perfusion [9]. Together, these defects can lead to retinal neurodegeneration that occurs in diabetes [10].

Usually, the evaluation of retinopathy is performed by examination of the eye fundus, but before the establishment of visible anatomical fundoscopic changes, the visual function was used as an indicator of underlying neuronal loss [11]. In addition, psychophysical procedures have been widely used to study the visual function of patients suffering from diabetes [12-17].

Visual acuity has been extensively used as a noninvasive indicator of visual function [18,19], and it is well known that it can be affected in diabetes. However, visual acuity changes cannot always be used to differentiate the visual function of eyes with and without established retinopathy [3]. Moreover, visual acuity measurements provide only limited evaluations of the subject’s spatial vision. Visual acuity is correlated with high spatial frequency cutoff of the luminance spatial contrast sensitivity function, and represents the highest spatial frequency that a subject sees at high contrast. As shown in previous studies, visual acuity can be preserved in some cases of diabetes, while at the same time intermediate and low spatial frequencies may show severe contrast sensitivity decreases [3]. In addition, it has been shown that retinopathy caused by diabetes increased the range of spatial frequencies with impaired contrast sensitivity [13].

Colour vision evaluation is another important tool used to monitor the visual function of patients with diabetes. Several colour vision tests are available, including measurements of the patient’s ability to perform colour arrangement, estimates of colour discrimination thresholds, and measurements of colour contrast sensitivity. The Farnsworth–Munsell 100 hue test (FM 100 test) is a colour arrangement test that has been used for colour vision evaluation in diabetes and other diseases that affect the visual system. Initial studies of diabetic patients showed high FM 100 scores, especially along the blue-yellow axis [4]. An investigation of the colour vision in 2701 patients enrolled in the Early Treatment Diabetic Retinopathy Study, using the FM 100 test, showed blue-yellow losses that were correlated in magnitude with the severity of macular oedema, but there were also colour vision losses in patients without macular oedema [20]. In a study of diabetic patients without retinopathy using another colour arrangement test, the Lanthony Desaturated D-15d, it was found that deficits were restricted to the tritan axis [21]. The colour discrimination ellipses of the same group of diabetic patients estimated with the Cambridge Colour Test (CCT) showed diffuse losses [21-23]. Diabetic patients evaluated by Colour Assessment and Diagnosis tests (CAD) also showed diffuse colour vision losses [24].

All these findings indicated that psychophysical evaluation of luminance contrast sensitivity and colour vision are important tools for the screening of visual function deficits in diabetic patients, but there are some aspects that need further clarification. Colour vision and contrast sensitivity impairment could be used to identify retinopathy at earlier stages. These two procedures should be further studied to determine their specificity and sensitivity for diabetic retinopathy evaluations. The mechanisms that support the results of both tests should be different. For contrast sensitivity measurements, the visual response is mediated by mechanisms working within threshold levels, while for colour vision evaluation estimated using the Farnsworth–Munsell 100 hue test, there are many additional mechanisms working at suprathreshold levels. Therefore, both tests could show different or complementary neural impairment.

It would also be important to establish the correlation between results obtained with psychophysical methods and information provided by metabolic markers. Previously, it was reported that although the investigations of contrast sensitivity and colour vision successfully differentiated nondiabetic subjects from diabetic patients without retinopathy, they were not adequate to separate patients with and without retinopathy [25].

In this study, we evaluated the luminance contrast sensitivity across a wide range of spatial frequencies and measured the ability of subjects to perform colour arrangements using the FM 100 test on a group of patients with type 2 diabetes, with and without retinopathy. We analysed the psychophysical results, and correlated these results with the levels of metabolic markers for the disease and with the time elapsed since disease diagnosis.

## Methods

### Subjects

We tested both eyes of 59 subjects, including 32 healthy subjects (64 eyes, 49 ± 12.3 years old) and 27 patients with type 2 diabetes (54 eyes, 53 ± 12.1 years old). In the type 2 diabetic group, 4 eyes of different subjects were later excluded from this study because they met the exclusion criteria that were applied, 20 eyes had nonproliferative retinopathy, and 30 eyes had no retinopathy. The criteria to diagnose retinopathy followed the international recommendations [26].

Subjects were recruited through the Program for the Control of Diabetes and Hypertension of the Health Basic Centre of the Federal University of Amapá, Macapá, Brazil. All procedures were approved by the Ethical Committee for Research in Humans of the Federal University of Amapá (Protocol #FR-278871/09).

All patients were interviewed by an endocrinologist regarding their health and clinical history. All patients were also examined by an ophthalmologist before starting the psychophysical tests. The ophthalmological exams included tonometry, visual acuity measurement, evaluations of eye refractive state, retinography, and fundoscopy. In addition, the Ishihara plates test was applied as a screening procedure to identify subjects with some degree of red-green colour vision loss.

Exclusion criteria included visual acuity of 20/40 or worse, congenital colour blindness, history of ophthalmological disease, advanced cataracts, and/or any chronic disease not associated with diabetes that could affect the visual system. None of the patients had previously received laser photocoagulation treatment.

### Psychophysical procedures

The contrast sensitivity test and the FM 100 test were implemented using an IBM desktop computer equipped with a graphics card. All stimuli were displayed on a 21-inch colour LCD monitor (spatial resolution of 1280 × 1200 pixels, 75 Hz, 8 bits, Ecofit P2270 model; Samsung, Seoul, South Korea) in a dark room. A dithering routine was used to obtain additional grey levels [27]. Luminance linearization was performed by gamma correction using a colorimeter (CS100-A; Konica Minolta, Osaka, Japan).

For the contrast sensitivity tests, the subject was positioned at a 3 m distance from the monitor. The contrast sensitivity was estimated for eleven spatial frequencies, 0.2, 0.5, 0.8, 1, 2, 4, 6, 10, 15, 20, and 30 cycles per degree (cpd), using vertical sinusoidal gratings in a field of 6° × 5° of visual angle and 43.5 cd/m^2^ mean luminance. The contrast threshold was estimated by using the method of adjustment starting from subthreshold contrast levels [28]. As the investigator increased the stimulus contrast, the subject’s task was to detect the grating presence on the screen and verbally inform the investigator. The contrast was then decreased until the subject reported that the stimulus was no longer detectable. This procedure was repeated 10 times, and the minimum perceived stimulus contrast was considered to be the contrast threshold. The contrast sensitivity was then estimated by the log_10_ of the inverse of contrast threshold value for each spatial frequency.

For the FM 100 test, the subject was positioned at a 1 m distance from the monitor. The stimulus was composed of 85 circles of different hues, saturated colours, 1° of visual angle, and 42 cd/m^2^ of luminance. The computerized test was equivalent to the original test illuminated by D65, and was previously applied in other clinical investigations [27,29]. Initially, the stimulus was shown to the subject as an ordered sequence of hues. Afterwards, the circles were mixed and randomly distributed throughout the monitor screen. The subject’s task was to rearrange the circles following a sequence of hues as similar as possible to what was previously shown. There was no time limit to perform the task. The errors in task performance were quantified and recorded as the log_10_ of the total score. The log-transformation in both the results of contrast sensitivity test and the FM 100 hue test were used to meet the assumptions of parametric statistical tests [27].

### Data analysis

The normative data of the psychophysical results were calculated by the tolerance intervals analysis with a 95% confidence level for 90% of the population. One- and two-way analysis of variance with a Tukey’s post-hoc test (with α = 0.05) were used to compare patients’ results with those obtained from control subjects. The results of the psychophysical evaluation were correlated with the time of the disease when it was initially diagnosed, fasting blood glucose level, and glycated haemoglobin level, by using the linear correlation method (with α = 0.05).

## Results

### Ophthalmological exam and evaluation with Ishihara colour plates

All subjects from the control group and type 2 diabetic group had normal trichromatic colour vision in the Ishihara plate test. Twenty out of 50 eyes were diagnosed with retinopathy, comprising 13 eyes with no proliferative retinopathy, six eyes with proliferative retinopathy, and one eye with retinopathy associated with macular degeneration.

Among the eyes from type 2 diabetic patients without retinopathy, five eyes had increased intraocular pressure (22 mmHg), six eyes had visual acuity worse than 20/20, and two eyes showed anatomical changes, including one eye with keratosis and one eye with papillary injury.

Among the eyes from type 2 diabetic patients with retinopathy, four eyes had increased intraocular pressure (30 mmHg), 10 eyes had visual acuity worse than 20/20, and six eyes showed anatomical changes, including five eyes with opacity of ocular media and one eye with iris atrophy.

### Clinical evaluation

The mean fasting blood glucose and glycated haemoglobin for the group of diabetic patients were 136.69 ± 56.81 mg/dl and 7.5% NGSP ± 1.19, respectively (NGSP: National Glycohaemoglobin Standardisations Program) [30]. The mean time elapsed since diabetes diagnosis was 6.69 ± 7.42 months. The mean time elapsed since diabetes diagnosis was similar between the type 2 diabetic patients without retinopathy (6 ± 7 months) and the type 2 diabetic patients with retinopathy (6 ± 7 months) (p = 0.22).

### Contrast sensitivity

**Figure 1:**
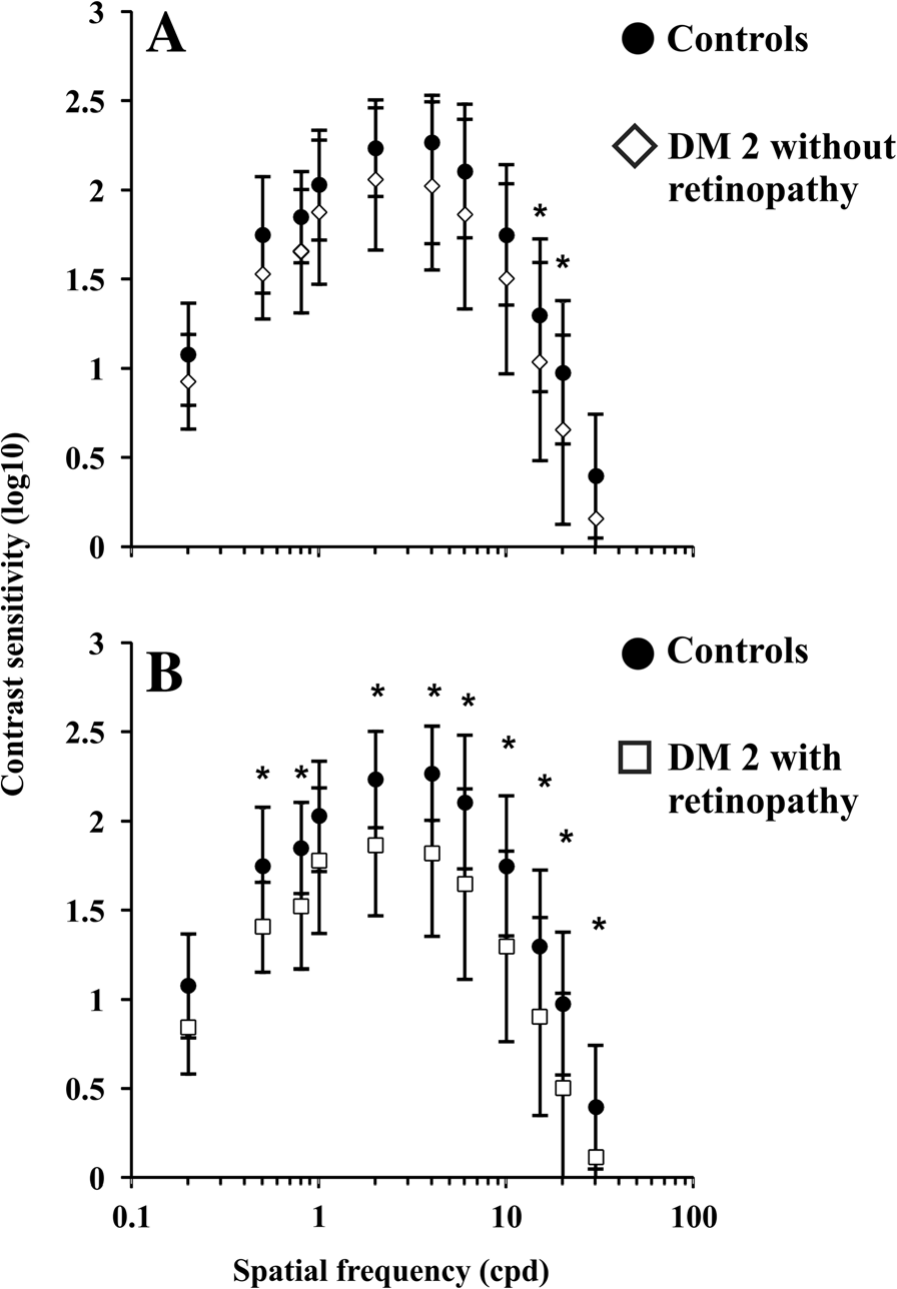
**Means and standard deviations for monocular luminance contrast sensitivity of controls and diabetic patients. (A)** Controls (filled circles) versus diabetic patients without retinopathy (empty diamonds). **(B)** Controls (filled circles) versus diabetic patients with retinopathy (empty squares). Controls had higher contrast sensitivity than diabetics in all conditions measured, but the level of significance (p < 0.05) was reached only in the conditions marked with asterisks (*). There was contrast sensitivity attenuation at 15 and 20 cpd for patients without retinopathy, and in the range between 0.5 and 30 cpd for patients with retinopathy. There was no statistical difference between the two diabetic groups in any spatial frequency. DM 2: type 2 diabetes.

Table 1 shows the range of tolerance intervals for the control group estimated from the contrast sensitivity test. Monocular luminance contrast sensitivity was lower than the normal tolerance limits in at least one spatial frequency in a large proportion of diabetic patients: 31 of 50 eyes from patients with type 2 diabetes; 13 of 30 eyes from patients with type 2 diabetes without retinopathy; and 11 of 20 eyes from patients with type 2 diabetes with retinopathy (Table 1).The group of diabetic patients without retinopathy had lower mean monocular contrast sensitivity at all spatial frequencies than the control group, but the difference only reached statistical significance in two out of 11 spatial frequencies tested, 15 and 30 cpd (p < 0.05). The group of diabetic patients with retinopathy also had lower mean monocular contrast sensitivity than the control group, at all spatial frequencies, but the difference was statistically significant at 0.5, 0.8, 2, 4, 6, 10, 15, 20, and 30 cpd (p < 0.05), which was nine of 11 spatial frequencies studied. Figure 1 shows means and standard deviations for contrast sensitivity at all spatial frequencies for the two groups of diabetic patients, and compares the results with values obtained from control subjects.

**Table 1:** Normal range for contrast sensitivity test and FM 100 scores estimated from a control group

	**Contrast sensitivity (log**_**10**_**)**											**FM 100 score (log**_**10**_**)**
	**0.2 cpd**	**0.5 cpd**	**0.8 cpd**	**1 cpd**	**2 cpd**	**4 cpd**	**6 cpd**	**10 cpd**	**15 cpd**	**20 cpd**	**30 cpd**	
**UTL**	1.61	2.34	2.33	2.59	2.73	2.75	2.81	2.49	2.09	1.71	1.03	1.88
**LTL**	0.53	1.1	1.31	1.45	1.73	1.78	1.39	0.96	0.5	0.2	0.23	1.73
	**Number of eyes with impaired results compared with the tolerance intervals**											
	**Contrast sensitivity (log**_**10**_**)**											**FM 100 score (log**_**10**_**)**
	**0.2 cpd**	**0.5 cpd**	**0.8 cpd**	**1 cpd**	**2 cpd**	**4 cpd**	**6 cpd**	**10 cpd**	**15 cpd**	**20 cpd**	**30 cpd**	
**DM2 NR**	2	1	4	4	5	11	9	5	6	9	0	26
**DM2 WR**	0	1	3	2	7	11	7	6	4	6	0	18

UTL = upper tolerance limit, LTL = lower tolerance limit, DM2 NR = type 2 diabetic patients with no retinopathy, DM2 WR = type 2 diabetic patients with retinopathy.

### Colour vision

**Figure 2:**
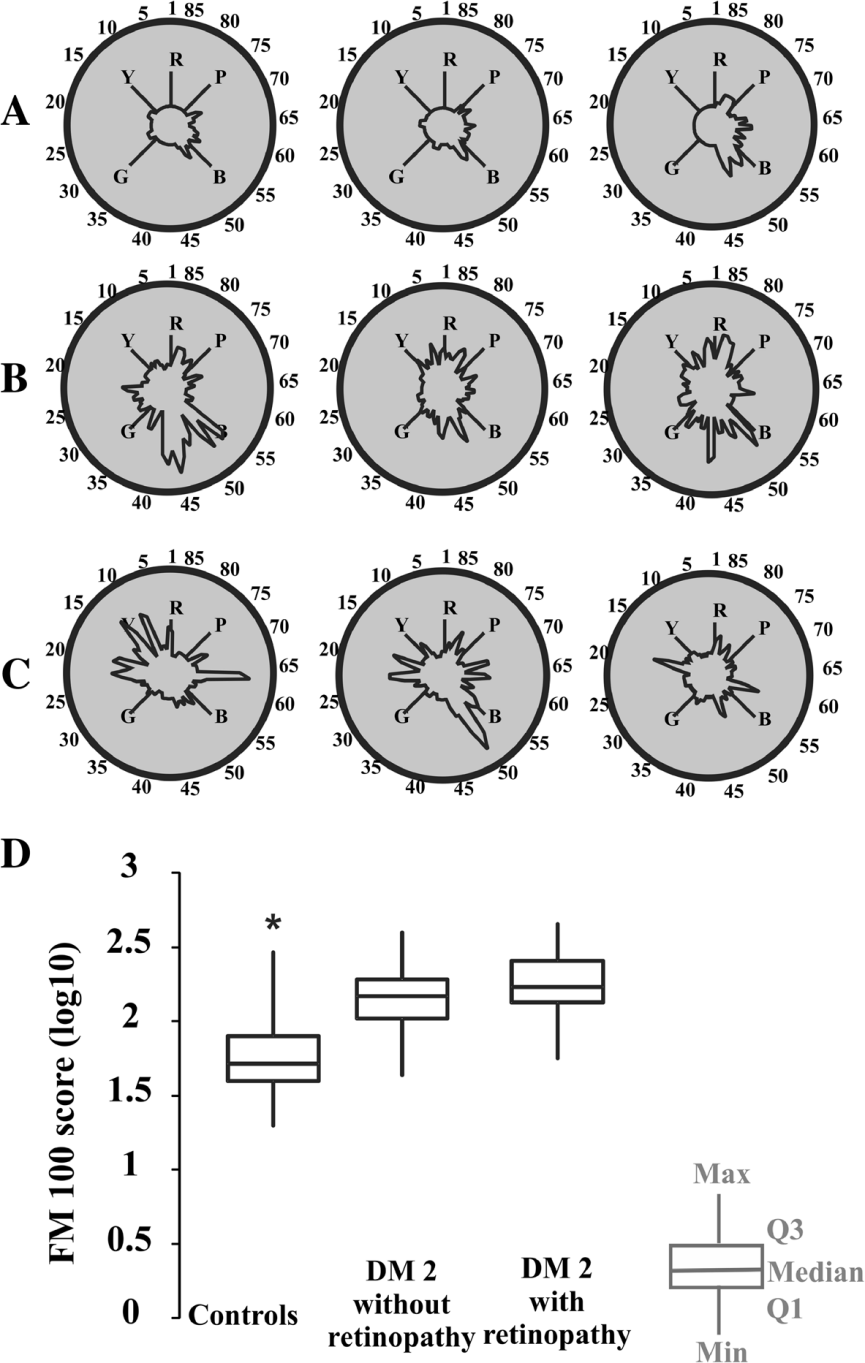
**Monocular colour vision of diabetic patients evaluated with the FM 100 hue arrangement test (A-C).** Individual results for three subjects of each group: **(A)** controls, **(B)** diabetic patients without retinopathy, and **(C)** diabetic patients with retinopathy. Diabetic patients made more mistakes than controls, but the errors were diffusely located in the FM 100 colour space without preference for any colour vision axis. **(D)** Box plots of the FM 100 error scores (log_10_ values). The control group made smaller numbers of mistakes than the two diabetic groups (p < 0.05). There was no difference between diabetic groups. DM 2: type 2 diabetes. Q1: First quartile. Q3: Third quartile. R = red, Y = yellow, G = green, B = blue, P = purple.

Table 1 shows the range of tolerance intervals for the control group, estimated from the FM 100 test. The ability of diabetic patients to perform the FM 100 hue arrangement test, using their monocular vision with either eye, was impaired in 44 out of the 50 eyes of type 2 diabetic patients tested, and they had FM 100 error scores above the normal tolerance limits (Table 1). The errors of diabetic patients were diffusely distributed across the FM 100 colour diagram without a specific colour axis preference. Figure 2A-C shows, respectively, the individual results for three controls, three patients without retinopathy, and three patients with retinopathy. The type 2 diabetic group without retinopathy had 26 eyes out of 30 with scores above the normal tolerance limits, while the retinopathy group had 18 eyes out of 20 with scores above the normal limits. Figure 2D shows the comparison of monocular performance in the FM 100 test between normal controls, diabetic patients without retinopathy, and diabetic patients with retinopathy. Both groups of diabetic patients differed from controls (p < 0.01) but were statistically similar to each other (p > 0.05).

### Correlations among the groups

There were moderate correlations between time of diagnosis and contrast sensitivity, ranging from 2 to 10 cpd for the eyes with no retinopathy, and at 0.8 cpd for the eyes with retinopathy. There was a moderate correlation between glycated haemoglobin level and contrast sensitivity at 0.2 cpd for eyes with no retinopathy, and at 0.2, 0.8, and 2 cpd for eyes with retinopathy. There was a correlation between fasting blood glucose level and contrast sensitivity at 0.2, 0.5, and 0.8 cpd for eyes with no retinopathy, but there was no correlation between fasting blood glucose level and contrast sensitivity at any spatial frequency for eyes with retinopathy. There was no significant correlation between time of diagnosis, fasting blood glucose level, and glycated haemoglobin level with FM 100 scores.

**Figure 3:**
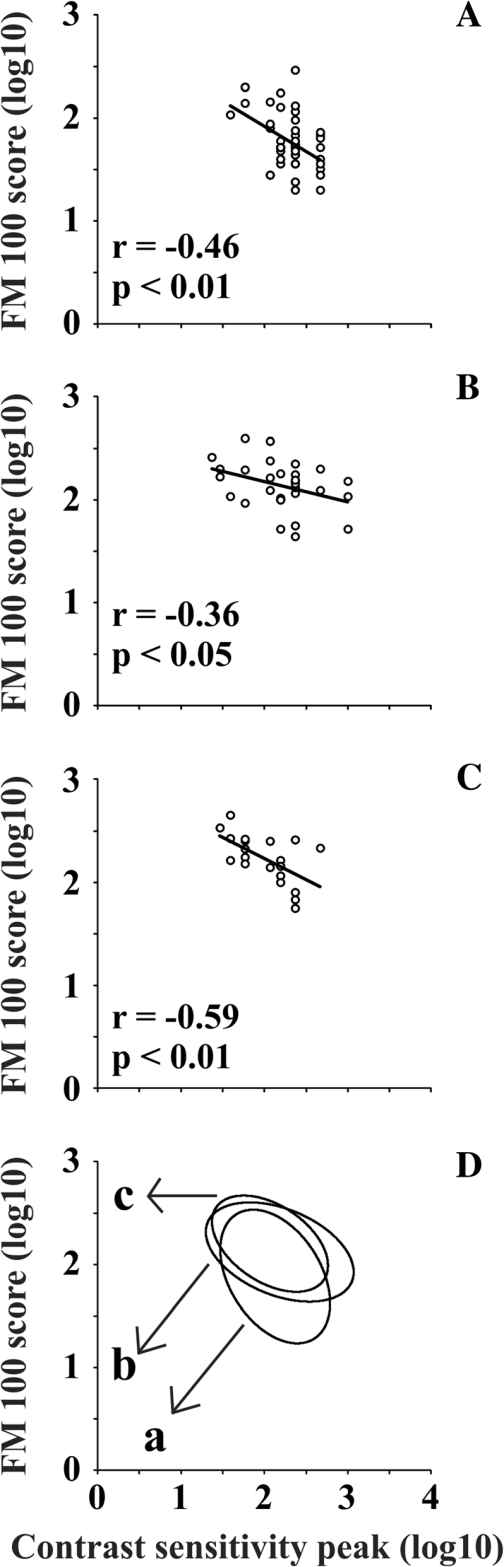
**Correlation between contrast sensitivity peak value and number of mistakes made in the FM 100 hue arrangement test.** Psychophysical tests were performed using monocular vision. Contrast sensitivity peak value and number of FM 100 mistakes were plotted using a log_10_ scale. **(A-C)** Data points were fitted with linear regressions using the least square method: **(A)** controls, **(B)** diabetics patients without retinopathy, and **(C)** diabetic patients with retinopathy. There were moderately significant linear correlations between the FM 100 score and CSF peak for the three groups of subjects, but correlation was higher for controls **(A)** and diabetic patients with retinopathy **(C)** than for diabetic patients without retinopathy **(B). (D)** Data points were fitted with ellipses using the Khachiyan Ellipsoid Method: (a) controls, (b) diabetic patients without retinopathy, and (c) diabetic patients with retinopathy. Diabetic ellipses are higher in the diagram than the control ellipse owing to their larger FM 100 scores. The ellipse from the diabetic patients with retinopathy is displaced to the left in the diagram in comparison with the ellipses from diabetic patients without retinopathy and from controls, owing to their lower contrast sensitivity peaks.

Figure 3A–C shows the correlation between the contrast sensitivity peak value (CSF peak) and the number of mistakes made in the FM 100 test (FM 100 score). We used the least squares method to obtain the regression straight lines across the data, expressed in log_10_ scales. There were moderate correlations for the three groups, which were higher for control subjects (r = -0.46, p < 0.01), and patients with retinopathy (r = -0.59, p < 0.01), and lower for patients with no retinopathy (r = 0.36, p < 0.05).

The Khachiyan Ellipsoid Method [31] was used to fit the best ellipses to the data points corresponding to controls (Figure 3A), diabetic patients without retinopathy (Figure 3B), and diabetic patients with retinopathy (Figure 3C). Then, the ellipses representing the three groups were compared as shown in Figure 3D. Diabetic group ellipses are higher in the diagram than control ellipses owing to their larger FM 100 scores. The ellipse from the diabetic patients with retinopathy is displaced to the left in the diagram, in comparison with the ellipses from diabetic patients without retinopathy, and from controls owing to their lower contrast sensitivity peaks.

## Discussion

It has been reported that psychophysical measurements are useful for monitoring the effects of diabetes on the visual system. Several studies identified visual losses of contrast sensitivity and colour vision associated with diabetes in the presence or absence of retinopathy, and used visual function as a biomarker to indicate the diabetic disease status [3,4,20-24].

In this study we analysed two commonly used visual tests to evaluate the visual functions of diabetic patients. Both tests have advantages and disadvantages that apply to diabetic patients. An important disadvantage is that both tests required long testing time periods and were very demanding in terms of the patient’s attention and commitment. These factors may have contributed to the data variability. However, the visual tests have been applied in several studies, and our results could be directly compared with previously reported results. Few studies have incorporated both tests and correlated each result for the same patient groups. We suggest that our findings were important because they showed that patients without retinopathy may develop visual function impairment even before the clinical symptoms appear, and that contrast sensitivity and colour vision results can be used as clinical measurements to identify the status of the visual function of the newly diagnosed diabetic patient.

As previously reported, we found that diabetic patients with retinopathy had worse results compared with patients without retinopathy and compared with control subjects. The visual impairment of eyes with retinopathy was due to retinal neurodegeneration caused by diabetes [32]. It was shown to be related to mitochondrial dysfunction, retinal capillary death, and formation of acellular capillaries [33,34].

Our results showed similar monocular luminance contrast sensitivity estimated from eyes without retinopathy and control eyes for the majority of spatial frequencies that were studied. In contrast, the eyes with retinopathy showed a decrease in monocular luminance contrast sensitivity throughout the entire spatial frequency domain. These results were similar to those of Sokol et al. [13], while other studies showed that, even in eyes without retinopathy, there was a contrast sensitivity decrease in a wide range of spatial frequencies [35,36]. We suggest that the differences in these results, when compared with others, may be due to differences in patient eyes and/or time from diagnosis.

For the investigation of luminance contrast sensitivity, we used sinusoidal gratings at several spatial frequencies as stimuli to detect the contrast threshold. Other studies with diabetic patients applied different stimuli configurations to estimate contrast threshold [25,37-40]. However, the use of sinusoidal grating was important because the visual system had different channels for spatial frequency processing. It is still not clear which channels would be impaired in diabetics. Different studies showed deficits in the contrast sensitivity estimated at different ranges of spatial frequency [14,16,41]. In diabetic patients, the reduction of the contrast sensitivity occurred even when the visual acuity was preserved [42,43].

In this study, the diabetic patients as a group showed higher FM 100 scores than controls, but the FM 100 test was not able to differentiate the diabetic group with retinopathy from the diabetic group without retinopathy.

The errors performed by diabetic patients, in the hue arrangement test, had no chromatic axis preference. There have been conflicting reports regarding axis preference in diabetic colour vision loss. Many studies that used the FM 100 test to evaluate the colour vision of diabetic patients reported preferential losses in the blue-yellow axis [4,20,44-50], but there were other studies using the same test that showed no colour axis preference [14]. In addition, diffuse losses were reported by measuring colour discrimination thresholds [22] or by evaluating individual colour spaces [51]. Studies also found that the colour vision of the same group of diabetic patients without clinical signs of retinopathy could be measured by hue arrangement ability with the D-15d test, and their colour discrimination thresholds could be determined with the CCT. Tritan axis losses were found in the hue arrangement test and diffuse losses were found in the discrimination thresholds [21]. Using CCT, it was found that colour discrimination losses were preferentially found within the protan axes in a group of diabetic patients without retinopathy [23]. In another study using the colour vision assessment (CAD) test it was observed that diabetic subjects exhibited equal and highly correlated reduction in red-green and blue-yellow thresholds [24]. It may not be possible to directly compare the results from FM100 with other tests such as CCT and CAD. The task in FM100 is performed at suprathreshold levels, while for CAD and CCT the final results are estimated for the threshold level. CAD and CCT investigate the functioning of cells or neuronal processing with highest sensitivity, while in the FM100 test it is possible that more cells or neuronal processes than those with high sensitivity may contribute to the patient’s performance.

Previously, other investigations used luminance sinusoidal gratings and FM 100 tests to estimate contrast sensitivity and colour vision of a group of diabetic patients [14,35]. Trick et al. [14] showed that type 2 diabetic patients without retinopathy, with little retinopathy, or with well-developed retinopathy had decreased contrast sensitivity in the intermediate range of spatial frequencies, and showed diffuse colour vision losses. They also reported that contrast sensitivity loss was more prevalent than impoverished hue discrimination among diabetic patients. Malukiewicz et al. [35] showed contrast sensitivity losses at intermediate and high spatial frequencies in type 2 diabetic patients. In addition, they evaluated patients’ colour vision by anomaloscopy and found colour vision losses, especially along the tritan axis.Because the interpretation of the contrast sensitivity test and the FM100 hue test results in diabetic patients with and without retinopathy are still controversial, we correlated the results of both tests in the same group of diabetic patients and in controls, to evaluate how the results changed. Data from control subjects and from patients with retinopathy had higher significant linear correlations in a two-dimensional space having FM 100 scores and CSF peaks as parameters, but data from patients without retinopathy were less significantly correlated (Figure 3). The main differences between controls and patients with retinopathy were that data from controls were located in the lower and middle regions of the diagram, while data from patients were located in the middle and upper regions of the diagram. The ellipses represented the area where most of the results for each group of patients could be found. We observed an overlapping area in the results from two-dimensional space, which we considered as an intermediate risk area for a newly diagnosed patient. The areas without overlap between the groups could be considered to indicate high (exclusive diabetic area) or low (exclusive control area) risk to develop visual loss due to diabetes. The results for patients without retinopathy suggested an intermediate level between controls and patients with retinopathy, indicating that the retinal damage was progressing, but differences could not be resolved using conventional ophthalmoscopy.

There has been no previous study showing the correlation between the FM 100 test score and contrast sensitivity peak for diabetic patients. This approach might be valuable for a variety of studies including those that measure contrast sensitivity by using methods other than sinusoidal gratings [17,52].

We found better correlations between the levels of metabolic parameters or time of diabetes diagnosis and monocular contrast sensitivity of diabetic patients without retinopathy, than among all the other parameters. However, there was no complete correlation between metabolic markers and psychophysical performance. Previous studies reported both positive and negative correlations between the level of metabolic markers and psychophysical parameters [14,17,24,44] while others found no correlation [53]. The variability of the clinical status from patients investigated across the different studies makes it more difficult to draw conclusions about the significance of these correlations. Higher values for time of diagnosis, glycated haemoglobin levels, or fasting blood glucose indicated poor control of glucose levels and this could be associated with a higher risk of vascular damage [54]. We found more significant correlations between contrast sensitivity and the metabolic markers from eyes without retinopathy. In addition, we found that the higher the value for metabolic markers, the worse the psychophysical performance. The impairment of glycaemic control alters neuronal function by both direct and indirect mechanisms. It is therefore possible that in eyes with retinopathy, the correlations between the visual performance and levels of metabolic markers could be nonlinear. Once the retinal damage occurs and the visual function is lost, glycaemic control may have less influence on the residual visual functions.

## Conclusions

Measurements of contrast sensitivity and colour vision ability of diabetic patients are important parameters to monitor how the disease progresses and how it impairs visual systems. Contrast sensitivity evaluations were useful to distinguish diabetics with and without retinopathy, but were not able to differentiate patients without retinopathy from controls. The FM 100 test was useful to distinguish diabetics from controls, but not diabetics with and without retinopathy. We also concluded that the use of two-dimensional spaces relating colour vision performance and contrast sensitivity might be useful to show the transition from normal vision to the vision of patients without retinopathy, and to the impaired vision of patients with retinopathy.

## Competing interests

The authors declare that they have no competing interests.

## Authors’ contributions

LCOA, EMCBL, MTST, LMO, and FPSP collected data and participated in data analysis. GSS contributed to the study design, participated in data analysis, and drafted the manuscript. ARR contributed to the study design and to computerized versions of the contrast sensitivity test and the FM 100 test. LCLS contributed to the study design and participated in data analysis. MIT led the study team, contributed to the study design, participated in data analysis, and drafted the manuscript. All authors helped to revise the manuscript as well as read and approved the final manuscript.

## Pre-publication history

The pre-publication history for this paper can be accessed here:

http://www.biomedcentral.com/1471-2415/14/104/prepub
